# FPA-FTIR Microspectroscopy for Monitoring Chemotherapy Efficacy in Triple-Negative Breast Cancer

**DOI:** 10.1038/srep37333

**Published:** 2016-11-18

**Authors:** Izabela Zawlik, Ewa Kaznowska, Jozef Cebulski, Magdalena Kolodziej, Joanna Depciuch, Jitraporn Vongsvivut, Marian Cholewa

**Affiliations:** 1 Laboratory of Molecular Biology, Centre for Innovative Research in Medical and Natural Sciences, Faculty of Medicine, University of Rzeszow, Warzywna 1a, 35-959 Rzeszow, Poland; 2 Department of Genetics, Chair of Molecular Medicine, Faculty of Medicine, University of Rzeszow, Kopisto 2a, 35-959 Rzeszow, Poland; 3 Department of Human Histology, Chair of Morphological Sciences, Faculty of Medicine, University of Rzeszow, Kopisto 2a, 35-959 Rzeszow, Poland; 4 Centre for Innovation and Transfer of Natural Sciences and Engineering Knowledge, University of Rzeszow, Pigonia 1, 35-959 Rzeszow, Poland; 5 Institute of Nursing and Health Sciences, Faculty of Medicine, University of Rzeszow, Kopisto 2a, 35-959 Rzeszow, Poland; 6 Monash Biomedical Imaging, Monash University, Clayton, Victoria 3800, Australia; 7 Institute of Nuclear Physics Polish Academy of Sciences, PL-31342 Krakow, Poland; 8 Australian Synchrotron, 800 Blackburn Road, Clayton, Victoria 3168, Australia

## Abstract

Triple-negative breast cancer is the most aggressive breast cancer subtype with limited treatment options and a poor prognosis. Approximately 70% of triple-negative breast cancer patients fail to achieve a pathologic complete response (pCR) after chemotherapy due to the lack of targeted therapies for this subtype. We report here the development of a focal-plane-array Fourier transform infrared (FPA-FTIR) microspectroscopic technique combined with principal component analysis (PCA) for monitoring chemotherapy effects in triple-negative breast cancer patients. The PCA results obtained using the FPA-FTIR spectral data collected from the same patients before and after the chemotherapy revealed discriminatory features that were consistent with the pathologic and clinical responses to chemotherapy, indicating the potential of the technique as a monitoring tool for observing chemotherapy efficacy.

Triple negative breast cancers (TNBCs) are a specific subtype of epithelial breast tumors that are negative for the protein expression of the estrogen receptor (ER), the progesterone receptor (PR) and lack overexpression/gene amplification of HER2 (hormone epidermal growth factor receptor 2)^1^. Approximately, 10–14% of breast cancers are triple negative subtype and because there is no known molecular change suitable for targeted therapy, this histological subtype is the most aggressive breast cancer subtype with limited treatment options and a poor prognosis^2^.

Current evidence for the cellular origins of breast cancer subtypes identified by different approaches including histology, molecular pathology, and genetic analysis was summarized and evaluated by Sims *et al*.^3^. Among physical techniques, mammography is the most commonly used method for diagnosing breast cancer^4^. Other techniques for detecting breast cancer include ultrasonography^5^, magnetic resonance imaging^6^, and positron emission tomography^7^. Optical techniques such as photo-acoustic imaging^8^ and fluorescence tomography^9^, are also used. Stains and labels to enhance contrast in microscopic tests have led to many important discoveries. All of these techniques, however, involve time-consuming and cumbersome procedures, and can also interfere with the actions of drugs or small metabolites, or may be cytotoxic. In contrast, label-free chemical imaging requires no artificial modification of biomolecules or additional sample preparation, and permits the comprehensive characterization of heterogeneous materials^10^. Vibrational spectroscopy including Fourier transform infrared (FTIR) absorption^11^ and Raman scattering^12^ techniques, provide molecular information of analyzed materials without perturbing the system under analysis. In particular, the strong FTIR spectral features make FTIR spectroscopy and microscopy a straightforward, non-destructive, label-free chemical identification modality with broad applications^11^,^13^. FTIR spectroscopic technique has been widely used in biologic and biomedical fields such as marine biology, cancer research, stem cells, live cell biology, Alzheimer’s disease, and more^13^.

The aim of the present study was to develop a diagnostic approach based on FTIR imaging technology and multivariate data analysis, specifically principal component analysis (PCA), to monitor the course of chemotherapy in seven female patients suffered from triple-negative breast cancer. Accordingly, we present here a FTIR-based approach that substantially extends the current capabilities of breast cancer diagnosis by providing insights into changes of biomolecular information in the breast tissue as a result of chemotherapy in different cancer patients. Such a knowledge will underpin further development of chemotherapy to achieve optimal efficacy in cancer patients with diverse biological makeups. It should be emphasized that our previous studies focused only on comparing healthy and tumor tissues collected from groups of patients. This study, on the other hand, is the first report of developing the combined FTIR and PCA approach for investigating chemotherapy efficacy within the same patients.

## Results

### Histopathologic examination

**Figure 1:**
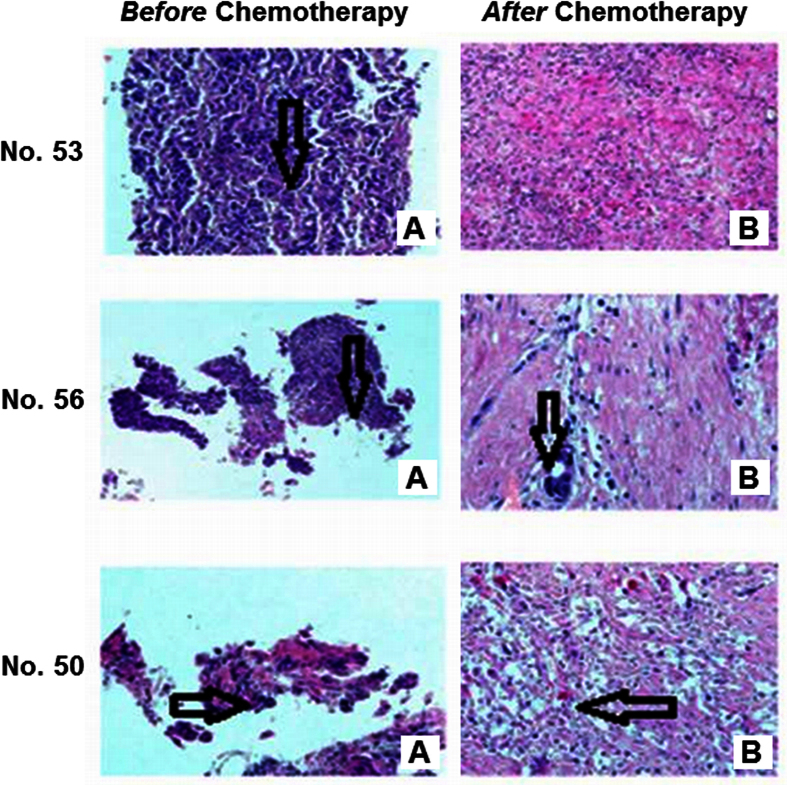
H&E images of breast cancer tissues obtained using tru-cut needle biopsy *before* (**A**) and *after* (**B**) chemotherapy. Arrows on images 53A, 56A and 50A indicate the presence of cancer tissue before chemotherapy. After chemotherapy, image 53B reveals a complete pathologic response after chemotherapy (i.e. no cancer cells detected in specimen). Image 56B represents the specimen after chemotherapy with partial pathologic response showing a presence of microfocus of residual cancer tissue, while image 50B indicates only minimal pathologic response evidenced by the residual cancer cells that exhibited increased cytologic changes and marked stromal fibrosis.

Histopathologic examination of the postoperative specimen of Patient No. 53 showed a complete pathologic response (pCR) after neoadjuvant chemotherapy, i.e. 100% of the cells in the hematoxylin and eosin (H&E)-stained slides were normal (ypT0N0). As illustrated in Fig. 1 (53B), only a fibroblastic focus of loosely organized connective tissue was detected with the accumulation of focal foamy macrophages, hemosiderin deposits, and mild inflammatory reaction. No lymphovascular invasion was observed.

The histopathologic study of Patient No. 56, on the other hand, revealed a partial pathologic response (pPR) after chemotherapy with a very small, yet detectable, amount of residual cancer tissue having a diameter of ~2 mm, as indicated in Fig. 1 (56B). Less than 10% of the cells were cancerous (ypT1aN0) that exhibited discernible nuclear alterations and dense interstitial fibrosis. Similar to Patient No. 53, no lymphovascular invasion was observed.

The histopathology of Patient No. 50 also demonstrated a pPR, but only at a minimal level because it was found that more than 50% of the residual cells were cancerous with multiple metastases to axillary lymph nodes (ypT2N2a). Residual cancer tissue in breast tumor and in axillary metastases exhibited increased cytologic changes and marked stromal fibrosis. Lymphovascular invasion was detected beyond the main mass of the tumor, as shown in Fig. 1 (50B).

Similar to Patient No. 56, the histopathological results of Patient No. 01 and 03 indicated pPR with residual cancer tissue found to possess the diameters of 6 and 0.15 mm for Patient No. 1 (ypT1bNx) and 3 (ypT1aNx), respectively. These further suggested that less than 10% of the cells in tumor after chemotherapy were cancerous. Dense interstitial fibrosis was also observed in these two patients. Although the result obtained from Patient No. 02 was also pPR and similarly less than 10% cancer cells was detected after chemotherapy with focus of cancer having the diameter of 4 mm, but the cancer cells found in this case clearly exhibited marked nuclear and cytoplasmic alterations (ypT1aN1a). In addition, a small metastasis was found in one axillary lymph node with nuclear alterations similar to those found in the main tumor.

The histopathologic examination of the post-operative specimen from Patient No. 04, on the other hand, was found to be similar to that observed in Patient No. 50 (ypT2N3a), indicating pPR with more than 50% of the residual cancer tissue and with multiple metastases to axillary lymph nodes (ypT2N3a). Cytological changes after chemotherapy were marked and stroma demonstrated fibrosis either in breast tumor or in metastatic foci in the lymph nodes.

### FPA-FTIR microspectroscopy

**Figure 2:**
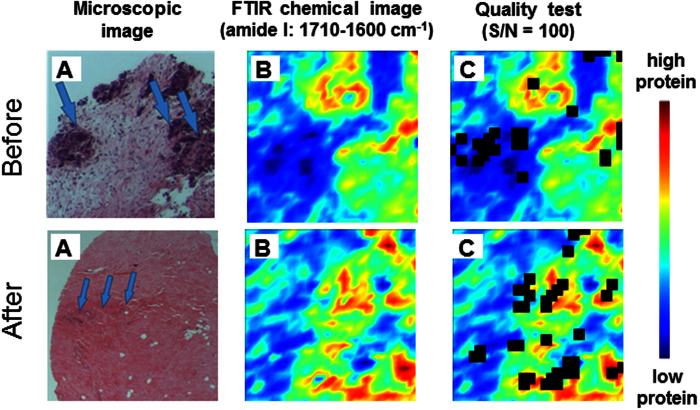
Schematic illustration of spectral pre-processing approach including (**A**) microscopic images of H&E stained sections used for selecting the regions of interest for spectral data collection, (**B**) FTIR chemical images of protein distribution using integrated area under amide I band (1710–1600 cm^−1^), and (**C**) their corresponding FTIR chemical images after quality test using S/N ratio criterion of 100.

As illustrated in Fig. 2, after completing histopathologic examination, FPA-FTIR microspectroscopic measurement was performed on the tissue sections that were microtomed in proximity to their corresponding H&E stained slides, so that the H&E images can be used to target the areas on the tissue where cancer and/or abnormal cells were present for the FPA-FTIR spectral data collection. The FPA-FTIR chemical image, as shown in Fig. 2B, was created based on integrated areas under amide I band (i.e. 1710–1600 cm^−1^), and thereby represent protein distribution on the examined tissue. The spectra composed in the FPA-FTIR image were then quality-screened using the signal-to-noise (S/N) ratio criterion as described in Method, in order to remove spectra of insufficient quality from the subsequent PCA calculation. As seen in Fig. 2C, the locations where spectra were excluded from the chemical image appear as black spots.

**Figure 3:**
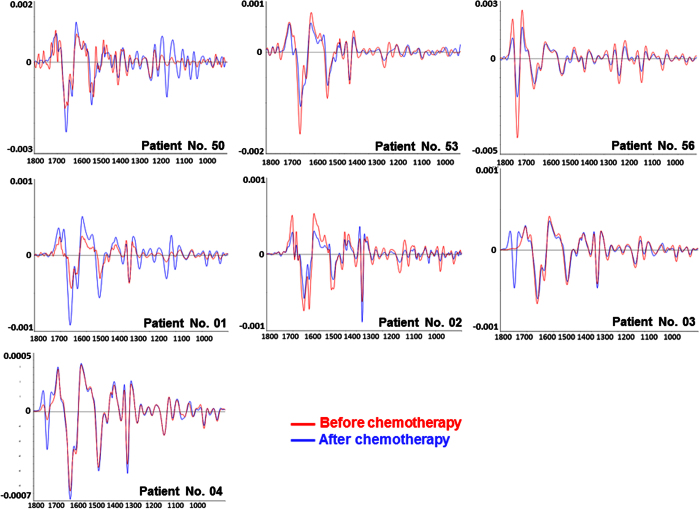
Representative EMSC-corrected 2^nd^ derivative spectra of the breast tissues collected from Patient No. 50, 53, 56 and 01–04 observed *before* and *after* chemotherapy.

At this stage, spectra that passed the quality-screening test were pre-processed through 2^nd^ derivatization and extended multiplicative signal correction (EMSC). The key aspects of using these two pre-processing methods and their advantages towards improved interpretability and enhanced performance of multivariate data analysis can be found in detail in Method section. After that, average EMSC-corrected 2^nd^ derivative spectra were calculated from each dataset to be used as representative spectra of breast tissues before and after chemotherapy for all of the seven patients, as illustrated in Fig. 3.

In an attempt to remove the contribution of paraffin bands from the analysis, the PCA was calculated based on two specific spectral regions of 1800–1500 and 1420–925 cm^−1^, in which bands associated to paraffin were absent. Accordingly, the spectral features within these two ranges contained biological bands of interest that were not subject to the interference from the paraffin used as an embedding agent. The detailed assignment of bands found in the spectra of the breast tissue in this study is based mainly on our previous FTIR studies of breast tissue^14^,^15^ as well as live marine microorganism^16^.

Based on the features of the average EMSC-corrected 2^nd^ derivative spectra in Fig. 3, the spectra are dominated mainly by protein bands attributed to amide I, II and III vibrational modes at 1650, 1545 and 1310 cm^−1^, respectively. Since the position of the amide I band observed in all patients was found to be within 1660–1650 cm^−1^, the majority of the proteins found in breast tissue was likely to be in forms of α-helix structure. In some cases particularly for Patient No. 56, the band at 1740 cm^−1^ assignable to ν(C = O) stretching vibration of esters was clearly observed. Since this band directly represents total lipids in the cells, it indicated that the tissue collected from Patient No. 56 both before and after chemotherapy likely possessed higher lipid contents than those obtained from other patients. Furthermore, the peaks at 1462 and 1411 cm^−1^ were also attributed to lipid composition inside the cells as a result of δ_scissor_(CH_2_) from methylene (-CH2) groups in acyl chains of lipid bilayers in orthorhombic packing and δ_rock_(CH_2_) of disubstituted *cis*-olefins^16^,^17^,^18^. In addition, the peak at 1049 cm^−1^ is associated with ν(C–O) coupled with δ(C–O) of C–OH groups of carbohydrates mainly from glycogen (Table 1).

**Figure 4:**
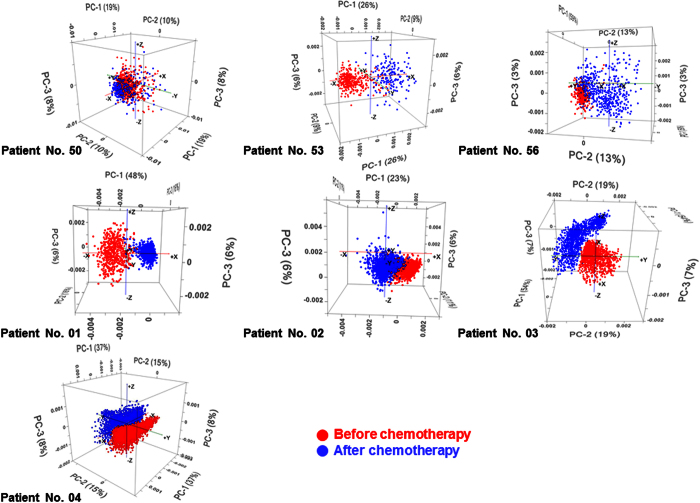
Corresponding PCA scores plot obtained using prior FPA-FTIR spectral data of the breast tissues collected from Patient No. 50, 53, 56 and 01–04 *before* and *after* chemotherapy, showing the discrimination patterns as a result of changes in biological compositions that were observed through FTIR spectral features in the regions of 1800–1500 and 1420–925 cm^−1^.

The PCA loading results revealed that the most influential bands leading to the discrimination pattern of breast cancer tissue before *versus* after chemotherapy, shown in Fig. 4, were those related to amide I and II bands, providing a molecular evidence that proteins are most susceptible to mutation during carcinogenesis^19^,^20^. However, there are also other strong bands found in the range of 1700–1300 cm^−1^, which possessed substantial contribution into the discrimination patterns. These bands can be attributed to various vibrational modes of the major macromolecules commonly found in biological samples^16^, which mainly involve proteins and lipids as well as their smaller units (i.e. amino acids and fatty acids, respectively).

### Discrimination by principal component analysis

PCA scores plots presented in Fig. 4 showed characteristic discrimination patterns of breast cancer tissue samples before and after chemotherapy, in seven different female patients. The distance between the two clusters in these PCA scores plot can be used to assess the chemotherapy efficacy.

In the case of Patient No. 53 for whom the treatment was found to be successful, the two clusters appeared to be well separated from one another, suggesting that the breast tissue after chemotherapy in this patient possessed significant changes in biochemical makeups compared to that observed before chemotherapy. On the other hand, the clustering pattern observed for Patient No. 56 appeared to be partly overlapped suggesting that the breast tissue after chemotherapy still exhibited biochemical features of the tumor tissue, which is consistent to the fact that the chemotherapy of this patient was found to be partially successful (i.e. <10% of cancerous cells detected).

Unlike the prior cases presented for Patient No. 53 and 56, the PCA scores plot observed for Patient No. 50 showed a distinct difference in which the two datasets were fully overlapped into a single cluster, strongly indicating a close similarity in biological makeups in the breast tissue before and after chemotherapy. Once again, the feature of the PCA scores plot was found to be consistent to the clinical observation, which revealed that the treatment for this patient was partially successful with >50% of the cancerous cells were detected after chemotherapy. Therefore, the fully overlapping feature of the PCA scores plot can be used as a supporting evidence to show that the majority of cells in the tissue after chemotherapy still possessed similar biochemical composition as those cancerous cells presented before the treatment.

## Discussion

In this study, the FTIR spectral features were characterized and assigned to specific biomolecules found in the breast cancer tissues collected before and after chemotherapy. This is the first report of using FPA-FTIR imaging technique together with PCA approach to investigate chemotherapy efficacy in triple negative breast cancer observed *within the same patients*. Insights into changes in biochemical composition obtained from the FTIR/PCA results, when correlated to the histopathologic results observed from the same patients, would lead to a better understanding at molecular level on how the certain type of chemotherapy treatment affects different individuals. Such a knowledge could underpin the development of new chemotherapy with enhanced efficacy for the treatment of breast cancer.

We previously reported a correlation between the FTIR spectral characteristics and the analyzed tissue (i.e. healthy breast tissue, cancerous breast tissue, and post-chemotherapy breast tissue). Our key finding was the correlation between the spectral characteristics and treatment efficacy^14^, which agreed well to one another suggesting that the key to the treatment response was in fact changes in biochemical makeups occurred in the tissue, which could be detected by FTIR and multivariate data analysis approach. We also found that, based on the observed FTIR spectral features, embedding biological tissue in paraffin did not alter chemical information in the analyzed materials, and hence was a suitable sample preparation for FTIR measurement^15^.

Our present results, on the other hand, revealed that FPA-FTIR microspectroscopy combined with multivariate data analysis (PCA) can be an effective analytical tool for monitoring chemotherapy efficacy. Although additional series of tests with a larger number of patients are crucially needed to draw a solid conclusion into the chemotherapy effect on different individuals, our current investigation based on FPA-FTIR and PCA approach clearly demonstrated that changes in biochemical composition in the breast cancer tissue observed after chemotherapy observed through FTIR spectral features coincide well with the state of health of the patients.

## Methods

### Breast cancer tissues

The study was conducted under Institutional Review Board (Protocol No. KBET/6/06/2014) from June 2014 at University of Rzeszow. The experimental protocols used in this study were approved by the institutional ethics committees (IECs) of the University of Rzeszow, and were carried out in accordance with the approved guidelines. Informed consents were obtained from all subjects.

The research was conducted on the basis of tissue materials obtained from seven triple-negative breast cancer female patients hospitalized in Subcarpatian Surgical Oncology Cancer Center in Brzozow between 2009–2016, treated for breast cancer with preoperative chemotherapy FAC (*5*-*fluorouracil, doxorubicin*, and *cyclophosphamide*) or AT (doxorubicin and docetaxel), followed by mastectomy. All triple-negative breast cancer patients were of white race with ductal type carcinoma. Patient No. 53 was 36 years old, grade G3 and cT2N1 (clinical staging before preoperative chemotherapy). Patient No. 56 was 51 years old, grade G3 and cT2N1. Patient No. 50 was 63 years old, grade G2 and cT2N2. Patient No. 01 was 31 years old, grade 3 and cT3N2. Patient No. 02 was 74 years old, grade 2 and cT2N2. Patient No. 03 was 54 years old, grade 2 and cT2N2. Patient No. 4 was 39 years old, grade 1 and cT2N1.

Clinicopathological characteristics of the breast cancer patients are presented in Table 2. For the FPA-FTIR microspectroscopic measurement, we used formalin-fixed paraffin-embedded (FFPE) breast cancer tissues obtained from the core biopsy of patients before and after chemotherapy. The FFPE breast cancer tissue were subsequently microtomed into 5-micron-thick sections and mounted on UV-grade CaF_2_ windows.

### FPA-FTIR microspectroscopic measurement

FPA-FTIR spectral images were acquired at Infrared Microspectroscopy (IRM) beamline, Australian Synchrotron (Clayton, Australia), using a Bruker Hyperion 2000 FTIR microscope (Bruker Optik GmbH, Ettlingen, Germany), equipped with a liquid-N_2_ cooled 64 × 64 element FPA detector and a 15× objective lens, coupled to a Vertex 70/70 v FTIR spectrometer (Bruker Optik GmbH, Ettlingen, Germany). Spectra were collected in transmission mode within a 4000–800 cm^−1^ spectral region as a single FTIR image covering a sampling area of 340 × 340 μm^2^. Each FTIR spectral image comprised a 32 × 32 array of spectra resulting from binning the signal from each square of 4 detectors on the 64 × 64 element FPA array. As a consequence, a single spectrum in each FTIR image represented molecular information acquired from *ca*. 10.6 × 10.6 μm^2^ area on the sample plane. For each tissue sample, high-quality FTIR spectral images were collected at 8-cm^−1^ resolution, with 64 co-added scans, Blackman-Harris 3-Term apodization, Power-Spectrum phase correction, and a zero-filling factor of 2 using OPUS 7.2 imaging software (Bruker). Background measurements were taken prior to sample spectral images, by focusing on a clean surface area of the CaF_2_ substrate using the same acquisition parameters. The areas on the FFPF breast tissue samples used for spectral acquisition were selected *via* visual examination on their corresponding H&E stained sections by targeting the area where traces of cancer and/or abnormal cells were observed, such as those shown in Figs 1 and 2A.

### Spectral pre-processing and multivariate data analysis

FPA-FTIR spectra embedded in each spectral image were first extracted and quality-screened using CytoSpec v. 1.4.02 (Cytospec Inc., Boston, MA, USA) by keeping for further analysis only the high-quality spectra with a minimum signal-to-noise (S/N) ratio of 100 measured over the spectral ranges of 1760–1600 cm^−1^ and 1900–1800 cm^−1^. These qualified spectra were then extracted and converted into a 2^nd^ derivative using Savitzky-Golay algorithm with 13 smoothing points. The advantage of 2^nd^ derivatization is essentially to eliminate the broad baseline offset and curvature as well as to enhance the features of hidden and overlapping bands, which is known to improve the performance of the multivariate data analysis by removing non-biological aspects such as optical and scattering artifacts from the input spectra^16^,^21^.

After that, the data were normalized using EMSC^22^,^23^,^24^. It should be emphasized that the EMSC algorithm is in principle a model-based pre-processing approach that corrects spectral artifacts commonly found in FTIR spectra of biological samples, including (*i*) additive baseline (or interference) effects and (*ii*) multiplicative scaling effects due to path-length differences, and effectively normalizes the spectra. As a consequence, the EMSC-corrected spectra without the spectral artifact components presents genuine chemically related (absorption) features that respond more linearly to the analyte concentration, thus resulting in a substantial improvement in interpretability and accuracy of the data in both qualitative and quantitative aspects.

Principal component analysis (PCA) was subsequently performed using The Unscrambler^®^ 10.1 software package (CAMO Software AS., Oslo, Norway). The EMSC-corrected 2^nd^ derivative spectral datasets of the tissue samples before and after chemotherapy from each patient were combined into one single set. PCA was subsequently performed on each combined dataset to investigate similarities and differences of biochemical makeups between tissues before and after chemotherapy. To exclude the bands associated with paraffin, the PCA was calculated using two spectral regions of 1800–1500 and 1420–925 cm^−1^.

## Additional Information

**How to cite this article**: Zawlik, I. *et al*. FPA-FTIR Microspectroscopy for Monitoring Chemotherapy Efficacy in Triple-Negative Breast Cancer. *Sci. Rep*. **6**, 37333; doi: 10.1038/srep37333 (2016).

**Publisher's note**: Springer Nature remains neutral with regard to jurisdictional claims in published maps and institutional affiliations.

## Figures and Tables

**Table 1:** Assignment of the major bands that possessed a significant influence on the PCA discrimination.

Wavenumber (cm^−1^)	Band Assignment
1654	amide I: α-helix
1462	δ_scissor_(CH_2_) from methylene (–CH2) groups in acyl chains of lipid bilayers in orthorhombic packing
1411	δ_rock_(CH_2_) of disubstituted *cis*-olefins
1049	ν(C–O) coupled with δ(C–O) of C–OH groups of carbohydrates mainly from glycogen

**Table 2:** Clinicopathological characteristics of breast cancer patients.

Case No.	Age	Race	Histological Type	Grade	Clinical staging before preoperaive chemotherapy	Preoparative chemotherapy	Pathological response	Type of surgery	Long-term follow-up
53	36	White	Ductal Carcinoma	G3	cT2N1	AT3s - 3 series Adria 85 mg, Taxotere 130 mg	ypT0N0	Mastectomia m.Madden	Alive without recurrence or metastases
56	51	White	Ductal Carcinoma	G3	cT2N1	FAC6s - 6 series Flu745mg, Adria 74 mg, CTX 745 mg	ypT1aN0	Mastectomia m.Madden	Alive without recurrence or metastases
50	63	White	Ductal Carcinoma	G2	cT2N2	AT6s - 6 series Adria 90 mg, Taxotere 160 mg	ypT2N2a	Mastectomia m.Madden	Death in 2 years after surgery, spine and lung metastases
01	31	White	Ductal Carcinoma	G3	cT3N2	AT6s - 5 series Adria 80 mg, Taxotere 150 mg	ypT1bNx	Mastectomia m.Madden	Alive without recurrence or metastases
02	74	White	Ductal Carcinoma	G2	cT2N2	AT6s - 6 series Adria 90 mg, Taxotere 160 mg	ypT1aN1a	Mastectomia m.Madden	Alive without recurrence or metastases
03	54	White	Ductal Carcinoma	G2	cT2N2	FAC 6 series 5Fu-650mg, Adria-65mg, CTX-650mg	ypT1aNx	Mastectomia m.Madden	Alive without recurrence or metastases
04	39	White	Ductal Carcinoma	G1	cT2N1	AT 6 series Adria - 75 mg, Taxotere – 115 mg	ypT2N3a	Mastectomia m.Madden	Alive without recurrence or metastases
